# High Risk Care Plans in Liaison Psychiatry

**DOI:** 10.1192/bjo.2022.466

**Published:** 2022-06-20

**Authors:** Yasmin Abbasi, Claire Jones, Marina Clark, Mohammad Meher Ali Chaudhry, Kit Akass

**Affiliations:** Surrey and Borders Partnership NHS Foundation Trust, Surrey, United Kingdom

## Abstract

**Aims:**

To audit completed liaison service high risk care plans against local and national guidelines.

**Methods:**

Sample comprised of a snapshot of all liaison patients currently on the case load on 14th December 2021. Electronic notes were reviewed to identify High Risk Care Plans (HRCPs) and audit completion against local guidance. Currently there is no national guidelines.

In addition staff from the liaison team were surveyed to consider their confidence in completing HRCPs in order to direct staff training. Acute hospital staff were also surveyed to ascertain positive and negative aspects of the current HRCPs, in order to suggest quality improvements ahead of the upcoming integration of new Digital notes system.

**Results:**

Sample size 284. High Risk Care Plans completed 11, with an additional 2 required but not found in the notes.

Non pharmacological deescalation advice was specified in only 2/11.

Regular medication was documented in 5/11.

Specialist rapid tranquillisation medication advice in 8/11.

8/11 made reference to the local rapid tranquillisation policy, which was not made available in the notes.

Absconsion risk is documented in 8/11 and advised level of observation 10/11.

**Conclusion:**

According to local guidelines High Risk Care Plans were appropriate for 4.6% of the liaison case load, but record was included in the notes for 3.9%. Of those completed mandatory fields including non pharmacological deescalation and rapid tranquillisation advice were not always complete. Reference to rapid tranquillisation policy not immediately available in the notes is largely unhelpful in an emergency.

Our local target is for 100% completion of appropriate high risk care plans and full documentation for each of the mandatory fields in the high risk care plan. Improved training and record keeping is required.

Staff survey suggested unfamiliarity with document and unclear boundaries between standard and patient specific information impaired utility of high risk care plans. We recommend familiarising staff with the document and encourage highlighted font for key information.

